# Alternative Reimbursement Models for Health Providers in High-Performance Sport: Stakeholder Experiences and Perceptions

**DOI:** 10.1186/s40798-023-00600-9

**Published:** 2023-07-11

**Authors:** Hannah E. Carter, Michelle J. Allen, Liam A. Toohey, Steven M. McPhail, Michael K. Drew

**Affiliations:** 1 Australian Centre for Health Services Innovation and Centre for Healthcare Transformation, Faculty of Health, School of Public Health and Social Work, Queensland University of Technology, 60 Musk Ave, Kelvin Grove, QLD 4059 Australia; 2 Athlete Performance Health, Australian Institute of Sport, Canberra, Australia; 3 University of Canberra Research Institute for Sport and Exercise (UCRISE), Canberra, Australia; 4 Digital Health and Informatics Directorate, Metro South Health, Brisbane, Australia

**Keywords:** Fee-for-service, Salary, Financial, Incentives, Pay-for-performance, Embedded provider, Health care, Economic, Outcomes

## Abstract

**Background:**

Value-based healthcare provider reimbursement models have been proposed as an alternative to traditional fee-for-service arrangements that can align financial reimbursement more closely to the outcomes of value to patients and society*.* This study aimed to investigate stakeholder perceptions and experiences of different reimbursement systems for healthcare providers in high-performance sport, with a focus on fee-for-service versus salaried provider models.

**Methods:**

Three in-depth semi-structured focus group discussions and one individual interview were conducted with key stakeholders across the Australian high-performance sport system. Participants included healthcare providers, health managers, sports managers and executive personnel. An interview guide was developed using the Exploration, Preparation, Implementation, Sustainment framework, with key themes deductively mapped to the innovation, inner context and outer context domains. A total of 16 stakeholders participated in a focus group discussion or interview.

**Results:**

Participants identified several key advantages of salaried provider models over fee-for-service arrangements, including: the potential for more proactive and preventive models of care; enhanced inter-disciplinary collaboration; and the ability for providers to have a deeper understanding of context and how their role aligns with a broader set of priorities for an athlete and the organisation. Noted challenges of salaried provider models included the potential for providers to revert to reactive care delivery when not afforded adequate capacity to provide services, and difficulties for providers in demonstrating and quantifying the value of their work.

**Conclusions:**

Our findings suggest that high-performance sporting organisations seeking to improve primary prevention and multidisciplinary care should consider salaried provider arrangements. Further research to confirm these findings using prospective, experimental study designs remains a priority.

**Supplementary Information:**

The online version contains supplementary material available at 10.1186/s40798-023-00600-9.

## Key Points

- There is evidence that value-based reimbursement models can incentivise healthcare providers to reduce over-servicing, potentially resulting in a more efficient use of scarce resources.
- Participants reported that salaried provider models conferred several key advantages over fee-for-service models, particularly in their ability to incentivise preventive and proactive care delivery.
- Our findings suggest that salaried provider arrangements have the potential to improve primary prevention and multidisciplinary care, when compared with fee-for-service arrangements. However, the design and implementation of reimbursement models should be informed by meaningful consultation with providers, athletes and other key stakeholders.

## Background

Healthcare providers in high-performance sport play a critical role in making decisions about the nature and type of treatment to provide athletes. However, little attention has been given to the ways in which different payment systems may be used to incentivise providers in these settings to deliver better quality, more efficient care.

Within health systems more generally, there has been increasing focus on the ways in which financial reimbursement models may be used to incentivise providers to reduce over-servicing, or to achieve pre-defined benchmarks of quality [1]. Often referred to as ‘value-based reimbursement models’, these systems provide an alternative to traditional fee-for-service arrangements in that they seek to incentivise the outcomes achieved from care, as opposed to the volume of services delivered [2]. A summary of commonly described health provider reimbursement models is provided in Table 1, along with their potential advantages and disadvantages in high-performance sport. In addition, alternative models may be designed using combinations from one of more of these core reimbursement structures, for example, a fee-for-service model combined with a fixed fee to account for time spent on non-consultation activities.

**Table 1:** Types of value-based reimbursement models and their potential advantages and disadvantages in high-performance sport settings

Type of model	Description	Potential advantages	Potential disadvantages
Fee-for-service	Providers are reimbursed separately for each distinct service provided	Simple to administer and enforce Ability to choose provider(s) No or limited management required of staff to the sporting organisation No on-costs of employment to the sporting organisation (e.g. leave loading, superannuation, long-service leave) Higher organisation control on what services are funded	May encourage over-servicing Can lead to fragmented/siloed care No or limited time allowed for coordination of care No incentives for prevention Providers may function independently rather than as a team Limited ability to design and implement prevention programmes Less autonomy in intervention choice for the provider
Pay-for-performance	Providers are financially rewarded for reaching key quality or performance benchmarks	Explicitly incentivises positive athlete outcomes Demonstrates commitment to evidence-based healthcare Transparent rewards process Can be used to focus attention on high-risk individuals/groups May provide a mechanism for healthcare to be aligned to organisational goals	No incentives to reduce unnecessary or low-value services or contain costs Can disincentive providers from seeing athletes with needs outside of targeted benchmarks Complex to establish and agree on evidence-based quality measures Can be difficult to measure outcomes in complex cases
Bundled payments	Providers receive a fixed, lump sum payment for a discrete episode of care for a given patient. Performance incentives are also commonly included in these models	Providers are discouraged from performing unnecessary or low-value interventions Strong incentive to avoid health-related complications Provider has flexibility to determine which services are offered to achieve the desired outcome	Can be difficult in sporting context to define what a discrete episodes of care would look like May encourage unnecessary episodes of care
Shared savings/risks	Providers earn bonuses and/or penalties based on spending below a predetermined benchmark over a period (typically contingent on meeting quality targets)	Providers are discouraged from performing unnecessary or low-value procedures Benchmarks can be determined and aligned to organisation strategy Incentivises activity towards health long-term health/performance outcomes if identified in benchmarks (e.g. low recurrence rates of injuries)	Up-front costs associated with developing the health IT and quality measurement infrastructure needed to reduce healthcare costs Assumes that providers are overspending and could penalise those already performing well May encourage ‘cherry-picking’ and other gaming behaviours May not be sustainable after initial savings have been realised May not account for the fluctuation in injury rates within sports each season due to external events (rule changes, weather etc.)
Organisational salary or contract-based provider engagement models	Providers are reimbursed based on agreed organisational priorities, regardless of the volume and type of services provided	Health services can be aligned with organisational priorities No incentives to provide low-value care Practitioners have one ‘boss’, reduced conflict between the sporting organisations goals and the healthcare providers/company goals Set salary/wage rather than market consultation fees	Need to manage staff performance Staff usually paid in salary, which may lead to complacency Staff on-costs need to be considered, e.g. superannuation, leave entitlements, backfill No incentive for performance typically Balance required for generalist vs specialist staff (with specialists typically being outside the in-house service)
Capitation	Providers are reimbursed on a per-person plan, regardless of the volume of services provided	Incentivises providers to keep athletes as healthy as possible through preventive care Incentive to keep costs per athlete low Encourages greater coverage by incentivising providers to take on more athletes	Providers are at increased financial risk which may not be practical to manage Can be complex to establish and enforce May lead to low care quality, particularly through under provision of care May encourage providers to select the healthiest/least complex athletes

The application of value-based reimbursement models in the high-performance sport setting has not yet been evaluated, thus their relative effectiveness is unknown. However, it is likely that these models would be of particular benefit within sporting organisations, where there is an overarching focus on maintaining athlete health and well-being through the prevention of illness and injury.

The aim of this study was to investigate stakeholder perceptions and experiences of different types of provider reimbursement models within the Australian high-performance sport setting. High-performance sport in this context encompasses Australia’s Summer and Winter Olympic, Paralympic and Commonwealth Games sports and athletes. Our findings may inform future planning and policy making for high-performance sporting organisations seeking to achieve effective and sustainable models of healthcare delivery.

## Methods

### Design

A pragmatic research approach was adopted, using purposive sampling of key stakeholders from a range of socioecological levels within the Australian high-performance sport system. Three focus groups and one individual interview were conducted between November and December, 2021. The study received ethical approval from the Australian Institute of Sport Human Research Ethics Committee (Approval Number 20211001). Participants provided verbally recorded informed consent before participating in the study. Patients and the public were not involved in the design or conduct of this research.

### Participants

Participants were purposively sampled and limited to individuals within the Australian high-performance system that had a direct stake and interest in the implementation of alternative healthcare funding models. Healthcare providers as well as management and executive-level personnel were included. The targeted sample size was between 12 and 18 participants to be recruited to one of three focus groups, chosen as a balance between pragmatic considerations and established guidance on focus group conduct [3, 4]. Purposive sampling of participants aimed to achieve a diversity of genders, health specialties, professional roles and sporting organisations. Participants were approached via an email from study investigators who were based within the high-performance sport setting.

### Data Collection

The use of focus groups was prioritised to stimulate engagement, interaction and discussion between participants, thereby stimulating ideas that might not have been uncovered in individual interviews [5]. Individual interviews were offered where participants were not available to attend any of the scheduled focus groups. Before attending the focus group/interview session, participants were asked to read a stimulus document containing general background information on value-based healthcare reimbursement models. The facilitator (author HC) also provided a summary of the study background and the key concepts at the commencement of each session, to ensure consistency in participants’ understanding and to define key concepts and terms.

All focus group discussions and individual interviews were conducted via a videoconference platform. Focus groups lasted between 96 and 148 min, while the individual interview lasted approximately 55 min. Written notes were taken during these sessions, with video and audio recordings, as well as verbatim transcriptions, also collected. Both facilitators also documented written summary notes immediately after the sessions had concluded.

All focus group sessions were jointly conducted by authors HC and MA, with HC conducting the individual interview. Author HC (PhD) is a health economist with content expertise and interest in value-based healthcare and provider reimbursement models. Author MA (PhD) is a qualitative researcher specialising in implementation science and is experienced in facilitating interviews and focus group discussions. Both facilitators were independent to the Australian high-performance sport system with no prior knowledge of, or connection to, study participants. No other individuals beside the participants and facilitators were present in the videoconference sessions. A member checking process was applied where participants were provided with a summary of the key findings from these sessions and had the opportunity to provide any additional comments, clarifications or feedback to the research team. No repeat interviews were carried out.

### Framework

A semi-structured question guide was developed based on the Exploration, Preparation, Implementation, Sustainment (EPIS) framework [6] and is provided in Additional File 1. The EPIS framework was selected as it allowed for a comprehensive and context-specific investigation of the potential challenges and opportunities of implementation value-based reimbursement models, which was being considered by decision makers within the Australian high-performance system. Consistent with this framework, questions were designed to investigate participants’ perceptions of how innovation factors (e.g. adaptability, characteristics and fit), inner context factors (e.g. organisational characteristics, individuals, knowledge, leadership) and outer context factors (e.g. funding, policy, networking) might impact on the implementation of alternative healthcare reimbursement models. The interview guide was flexible, allowing the facilitators to follow up and explore relevant themes raised in the discussion.

### Analysis Methods

Audio and written records from the focus group discussions and individual interview were subject to deductive thematic analysis by using an iterative and pattern matching approach in mapping to the relevant EPIS constructs [6, 7]. Author HC initially coded the verbatim transcription data and provided summaries of key codes and associated quotes. Author MA reviewed the analysis, listened to the audio recordings, as well as reviewing notes and quotes taken independently, then built on and refined the codes and themes. Both analysts coded based on a codebook with deductive EPIS constructs, as well as any inductive codes not captured in the EPIS framework. Both analysts then met and reviewed and refined the codes and themes further. The use of coding via audio recordings as part of pragmatic rapid analysis techniques is described further in Neal et al. [8] and Nevedal et al. [9], and has been shown to be equally as effective as more traditional qualitative analysis methods.

Quotes that were illustrative of key themes are reported verbatim with participants assigned a unique study number and identified as belonging to one four categories based on their current role: health providers; lead health providers (i.e. providers with national-level leadership roles within their disciplines); health managers; and sport managers.

## Results

A total of 16 stakeholders participated across three focus group discussions and one individual interview. A summary of participant characteristics is provided in Table 2. Participants were balanced across gender and there was a relatively wide range of roles represented. This included: healthcare providers in high-performance sport settings (*n* = 6); national lead providers within their respective disciplines (*n* = 2); health management personnel (*n* = 4); or performance management personnel (*n* = 4). Health provider participants included dieticians, physiotherapists, psychologists and medical doctors. Management staff included performance managers, a sports coordinator, health managers and executive personnel. Participants had experience working across a wide range of sports including cycling, diving, athletics, volleyball, triathlons, swimming netball and rowing. Three participants indicated they would like to participate but withdrew prior to attending their scheduled focus group session due to scheduling conflicts or competing workload demands.

**Table 2:** Participant characteristics

Participant characteristics	*N*	%
Male	8	50
Stakeholder type*		
Health providers		
Dietician	3	19
Sports and exercise physiotherapist	1	6
Sports and exercise physiologist	2	13
Psychologist	2	13
Sports medicine physician	1	6
Managers		
Health managers	2	13
Performance manager	4	25
Sport coordinator	1	6
Chief Executive Officer	1	6
Experience with provider reimbursement models*		
Fee-for-service	9	56
Salary-based (embedded provider)	14	88
Contracted daily rate	2	13

*Proportions may sum to more than 100% as individuals can be included in multiple categories

All participants involved in the discussions had some experience across either, or both, salaried and fee-for-service models within the Australian high-performance sport setting. There was limited experience across the cohort with other types of reimbursement models including pay-for-performance, risk-sharing or bundled funding arrangements. The discussions, therefore, were largely focussed on the key points of difference between fee-for-service and salaried provider models. Several participants referred to salary-based positions as ‘embedded providers’ in reference to the nature of these roles where providers are either full- or part-time employees of a particular sporting organisation, and whose role typically includes additional non-consulting activities such as coordinating care with other health providers and attending competitions, training sessions and team meetings.

Several key themes were identified from the discussions; these were analysed deductively and mapped as either ‘opportunities’ or ‘challenges’ to the corresponding EPIS domains of: (1) innovation; (2) inner context; and (3) outer context.

### The Innovation

#### Opportunities

##### Potential for Proactive Care

There was broad agreement among participants around some key opportunities of embedded provider models, in comparison with fee-for-service models. Participants noted the potential for these models to be more proactive in nature, with a greater focus on preventive care. The potential for financial benefits of prevention was also discussed, with several participants providing examples of instances where they believed the provision of primary prevention activities had contributed to reduced service use at a later stage. There was acknowledgement that individual disciplines were at different stages in terms of implementing preventive approaches, with mental health and nutrition seen as leading the way.

When athletes have easy access to services, they will be proactive… rather than sit on issues, wait for a formalised appointment and then catch the issue too late, then there’s a lot of training missed and big implications.

Health provider #1

##### Inter-Disciplinary Collaboration

The potential for embedded provider models to enhance inter-disciplinary collaboration was a dominant theme to emerge across all focus group discussions. It was noted that health problems in this setting, particularly as they relate to performance, are typically complex and cross multiple disciplines, meaning that collaboration is often necessary to resolve issues optimally. The importance of the distinction between multidisciplinary and inter-disciplinary collaboration was highlighted:

Instead of going around a medical room: update, update, update; its, let’s look at this athlete and how are we going to get them 3% faster, stronger, whatever the performance challenge is. Put the [performance] problem in the middle and we all come together collectively to solve the problem. When I’ve worked in organisations who transition to truly doing that, you get huge performance gain.

Lead health provider #1

The ability for inter-disciplinary collaboration to assist with breaking down barriers between disciplines was also highlighted. Participants described the benefits of this collaboration as not only relating to the level of expertise being contributed by those in specific fields, but the nature of the process in getting people to be comfortable in hearing other views that they may not have considered, or that may be in opposition to their own view but shared in a way that works towards finding an optimum outcome, with all relevant information being considered.

To break down some of those barriers, you need people to trust each other and be face-to-face, and that takes time out of consulting hours, but it’s incredibly valuable and you can really gain some enormous performance benefits over time with that approach.

Sports manager #1

Some participants described the flow-on effects of inter-disciplinary collaboration on athlete engagement. Specifically, athletes who observed the process of this collaboration within the context of an inter-disciplinary consultation or assessment were observed to have a greater appreciation for the complexity of treatment decisions, and the level of time, expertise and organisational resources that were behind these decisions.

You get greater adherence, it increases an athlete’s role and ownership of it, and confidence in the process when they understand the context.

Health provider #2

The importance of collaboration across both clinical and non-clinical staff was described. Participants perceived a shift away from the belief that the role of health providers was solely to influence health, while the role of performance coaches was to focus solely on performance. There was a general recognition of the significant influence of performance coaches on health outcomes, as well as clinician impacts on performance outcomes. The ability for embedded models of service delivery to promote relationship development between clinical and performance staff was suggested to be a key factor in facilitating this type of collaboration.

My experience with high-performance coaches is for the most part they are very relational people, and are often reluctant to engage in support without a sense of the person they’re working with, their motivations, their commitment to the programme.

Health manager #1

##### Understanding Context

The ability of embedded models to allow providers to achieve a greater sense of context was noted as being a key opportunity, relative to fee-for-service arrangements. This includes a deeper understanding of what an individual provider’s role was and where that was situated within the broader high-performance strategy for the athlete and the sport. This understanding of context was perceived to increase provider buy-in by providing a sense of purpose and shared goals.

The more embedded model allows the opportunity to understand far better what’s trying to be achieved with the athletes.

Health provider #3

##### Duty of Care Considerations

The duty of care providers felt for athletes in high-performance sport was described as being more involved than what typically exists in a private practice setting. The increased duty of care was attributed to the additional complexities in sporting environments. For example, providers are often responsible managing an athletes’ health while they are travelling, as well as having responsibility for whole teams of individuals, where issues affecting one individual may also impact on the broader team. By achieving cohesion and integration across all relevant aspects of healthcare, particularly for mental health issues, athlete outcomes are more likely to be optimised.

If we don’t have all the relevant information, we’re taking really big risks and practitioners can make naive decisions.

Health provider #2

##### Creating Incentives for Proactive Care

While it was generally recognised that embedded models had greater implicit incentives for activities such as proactive care and inter-disciplinary collaboration, some participants reported on their experiences in creating explicit incentives to further encourage these activities within both fee-for-service and embedded provider models. This included the provision of ‘billable’ fee-for-service time for non-consultation activities such as attending meetings, gym sessions or performing administrative tasks (e.g. shared care plans), as well as a formal directive for embedded providers to allocate a certain proportion of their time to non-consultation activities.

I’d rather there’s less consulting time, but the consulting that’s done is good quality because there’s a communication with other practitioners, coaches and sports and conditioning staff.

Health manager #2

It was noted that good integration can be achieved with external fee-for-service providers, but this continuity needs to be prioritised and resourced. An example was provided of a long-term fee-for-service provider who regularly attended competitions, travelled in camps and participated in other activities beyond the traditional clinic-based model. As such, the provider was able to gain a better understanding of the demands of the sport and what coaches needed, as well as allowing the athletes to view the provider as a core member of the team.

#### Challenges

##### Capacity of Providers to Deliver Proactive Models of Care

A perceived challenge to the successful implementation of embedded provider models was a lack of provider capacity. When providers were required to deliver services across a relatively large number of athletes on a limited full-time equivalent (FTE) allocation, their ability to deliver high quality and proactive or preventive types of care was likely to be diminished.

It’s more about being able to get access. Access to that expertise, being able to get a management plan continued throughout each athlete’s progression. They can’t be done if we have only 0.1 FTE across more than 40 athletes.

Sports manager #2

##### Attracting High Calibre Providers

The challenges of attracting and retaining highly experienced providers was highlighted as a key barrier to the successful implementation of embedded models. This arises from the disparity in provider remuneration levels available within the government funded sport system, in comparison with professional sports or private practice where providers can receive substantially greater remuneration. It was suggested that it may not be economically viable for experienced providers to be engaged on a full-time basis within government funded sport settings, with most opting to supplement their income through private practice.

There’s only so much you can do… I have to keep enough private work so I can support working in high-performance sport

Health provider #4

We’ve found that 0.4 [FTE] seems to be the sweet spot... you’re embedded enough to have a meaningful impact and do some proactive service delivery, maybe up to 0.6 [FTE]. Anything beyond 0.6 [FTE] you then lower the calibre of the provider, is what we’re finding.

Lead health provider #1

##### Lack of Specialist Expertise

Issues around generalisation versus specialisation as they relate to provider reimbursement models were discussed. It was acknowledged that embedded models are not able to achieve the level of specialist expertise available from external referrals to fee-for-service providers. Decisions, therefore, need to be made about which services to embed and which need to sit outside of that model and can be accessed on a needs basis.

It’s not one size fits all… we need to have the flexibility within the service to enable us to bring in the experts and specialists when required.

Lead health provider #2

While the lack of specialisation was a commonly perceived limitation of embedded models, the trade-off that comes with this was also acknowledged, with inter-disciplinary collaboration and coordination being prioritised over higher-end expertise on an acute basis.

##### Other Types of Reimbursement Models

In addition to fee-for-service and embedded models, there was some discussion around the potential merits and drawbacks of other types of arrangements. While none of the participants had direct experience with pay-for-performance models, some commented that these types of arrangements were unlikely to be effective in the context of high-performance sport. One participant noted the more common use of these arrangements within professional sport, where there was a perceived higher rate of ‘low-value care’ provision. Another participant suggested that pay-for-performance arrangements had the potential to be influenced by personal relationships and a provider’s networking ability, rather than outcomes. The potential for cultural issues to arise was also mentioned.

Culturally that would be difficult within the organisation. The organisation may struggle if different providers were engaged on entirely different arrangements.

Sports manager #1

Some providers discussed being engaged on a ‘daily rate’ or ‘retainer’ type arrangement, defined by the provision of a certain number of hours or level of access to the provider. There were mixed experiences under these arrangements. One provider indicated that the level of services they provided far exceeded the agreed number of days they were being reimbursed for, while another provider felt comfortable that they could provide an adequate service within the agreed terms.

I’m engaged for one and a half days a week, but I’ve tracked my time and it’s way more… it sits more around two and half days’ worth of hours

Health provider #5

I’m willing to take the risk… they can sign up for unlimited access to me, and I take the risk that I’m good enough at my job that they don’t ring me 10 times a day.

Lead health provider #1

### The Inner Context

#### Opportunities

##### Perceived Ability to Achieve Economic Efficiencies

Factors that were perceived to support the adoption or success of embedded service models included the assumption that these models represented better value for money from an organisational perspective. This was largely due to the increased focus on preventive measures.

Primary prevention we want to be our first line of defence, with tertiary prevention or intervention to be our last line of defence. In a fee-for-service model, it is near impossible to focus on any primary prevention, and very limited capacity for secondary prevention or early detection and management… We’re paying a lot of money to basically get tertiary prevention at best.

Health manager #3

##### Role of Internal Advocates

The key role of internal advocates for different reimbursement models was widely agreed to a key enabling factor. It was suggested that non-clinical management and executive roles, as well as athletes, would be particularly effective advocates. The important role of organisational leaders, as well as providers, in taking on an education role to increase the health literacy of coaches and athletes was also recognised as being an important driver of behaviour change in enabling a shift to more preventive care approaches.

Non-clinical, executive team members within large organisations play a fairly significant role as patrons and defenders of a more integrated healthcare system.

Health manager #1

Ultimately the athletes [should be advocates] as the users of the healthcare services… in a perfect world, you’d have the athletes with enough of a degree of their own health literacy to be able to ask questions and drive systems approaches... I don’t think that currently exists.

Health manager #2

##### Well Defined Service Agreements

The importance of clearly defined agreements and expectations when engaging providers on salaried models was noted. When providers lack this clarity, they may be perceived as less effective.

Being embedded requires real clarity in what that looks like… ambiguity is the enemy.

Lead health provider #1

##### Use of Data-Based Approaches

The potential for data-based approaches using injury and illness surveillance to identify problems was highlighted. For example, population level National Sporting Organisation (NSO) data could be used to identify key issues and develop and evaluate an intervention to target these. One participant described their experiences with using this approach to achieve a more efficient use of resources when the available funding was not sufficient to service the number of athletes they had responsibility for. They used internal injury surveillance data to identify programmes with the highest injury rates, and subsequently put targeted primary prevention interventions in place. This in turn brought injury rates down and allowed for additional provider time to be freed up to focus on other areas of need.

##### Effective Implementation

Factors that would likely contribute to the successful transition to different funding models were discussed. Participants highlighted the importance of: getting ‘buy-in’ from multiple stakeholders, both internal and external; allowing sufficient time for stakeholders to consider the proposed changes and contribute to their development; and adopting an iterative approach with changes introduced in phases. The adoption of a change management framework was recommended.

It takes some time to get everyone as close to being on the same page as possible… it’s something that can’t be done overnight.

Sports manager #2

#### Challenges

##### Ability to Demonstrate the Value of Services

A common and consistent theme across the discussions was the difficulty in defining and quantifying ‘value’ of service provision in a way that wasn’t directly linked to activity-based measures.

What is asked for from higher up management is how many consultations have occurred… [but] it does not give an appropriate measure on the quality of service that’s being provided.

Health manager #4

The difficulty in defining and measuring positive health as a concept was discussed. This leads to health often being framed in a negative sense, for example, the absence of illness or injury. This in turn makes it difficult to understand and quantify how optimal health in a positive sense may relate to better performance outcomes.

It’s really hard to measure the absence of an event. I can measure a physio appointment, I can measure a psych consult. But an athlete going: I’m psychologically really clear, focussed, and know what I’m going to do so I don’t need [a psych consult] because we’ve done the work, how do you measure that? How do you actually track that in [Athlete Management System] AMS? That’s where we’ve ultimately got to get to if we’re truly saying we’re doing proactive service delivery.

Lead health provider #1

Some participants had experiences with using process-based measures to track and measure provider impact. This included the implementation of periodic health evaluations, medical reviews or provider-specific health management plans that could then be appropriately actioned and followed up. The importance of promoting an athlete-centric approach was also recognised.

##### System-Level Barriers

Several system-level barriers to embedded provider models were identified. A misalignment in approaches was described around the role of mental health services, which the national high-performance system directly resources via external referral, while some individual sports and state institutes instead advocate for fully embedded psychology service provision.

Other system-level barriers were identified around the ways in which providers are expected to report on their services provided using internal athlete management systems, which can be at odds with a preventive, value-based approach.

You get paid for preventing having to pick up pieces, yet we have a system that wants to track us picking up pieces.

Lead health provider #1

##### Top-Down Decision-Making Processes

A common theme that emerged as a potential challenge to the implementation of alternative reimbursement models related to top-down decision-making that did not consult with relevant experts or stakeholders. There was a perception that decision-making was not always informed by evidence-based practice, including the use of national or international clinical guidelines. It was also suggested that decision makers should be better leveraging the relevant expertise within the organisation.

Additional concerns were raised about the role of management in imposing restrictions on providers and organisations that limit their autonomy, in turn undermining the key benefits of embedded provider models. This included placing onerous requirements on providers to demonstrate arbitrary measures of activity, the imposition of strategies such as voucher systems, and overly rigid requirements about the level and mix of services that can be purchased within the allocated funding.

Management gets involved to try and quantify service and then restrict it, they might introduce voucher systems, and then it’s almost like a fee-for-service model within an embedded model, and it just doesn’t work.

Sports manager #3

##### Lack of Appropriate Health Service Coordination

Participants described the challenges that can arise in terms of coordinating and managing a group of health professionals. There is often no dedicated role for this, and it is often left to the coach to receive and filter all relevant information. Further, when providers want to recommend a treatment option that is classified as discretionary in nature, the budget often sits within the sport, and therefore, puts coaches or administrators in the position of deciding what medical treatment they will pay for. Participants did not believe that coaches were best placed to perform this role, as they typically lacked the necessary skillset and expertise, and their role has multiple competing, and potentially conflicting, demands. This is particularly an issue with less experienced coaches who may not be used to working in a high-performance sport environment.

Some coaches, it’s almost too much for them to deal with a sports scientist or a physiologist at times if they just don’t understand it, and how they can best utilise those services.

Sports manager #1

The role of a ‘sports science and sports medicine coordinator’ was mentioned as a potential solution to these issues; these roles have been largely discontinued in recent years due to the lack of available funding. Other alternatives that were suggested included the use of a small reference group of two or three individuals with a broad combined knowledge base spanning both health and performance, that could make decisions or provide guidance.

### The Outer Context

There was relatively little discussion around the impact of outer context factors as either opportunities or challenges to the implementation of provider reimbursement models. The increased availability of funding into the high-performance sport system, of the back of Australia’s recently announced successful Olympic bid, was mentioned as a key opportunity for making changes to service provision models.

[The Australian Olympic bid] provided some options to consider that may not have necessarily been possible at a previous resourcing level.

Health manager #1.

An additional outer context factor mentioned was the International Olympic Committee position statement on athlete mental health, in particular its acknowledgement of interdependencies that contribute to both athlete mental health and health outcomes. This was an example of international-level recommendations driving the approaches being adopted within Australian sporting organisations [10].

## Discussion

This study investigated stakeholder perceptions and experiences of value-based healthcare provider reimbursement models within the Australian high-performance sport setting. Reflecting the experiences of the participants, these discussions focussed largely on the differences between fee-for-service models and salaried (“embedded”) provider models. Participants identified both challenges and opportunities associated with each of these models, with important implications for sporting organisations seeking to maximise value from limited healthcare resources.

### Policy and Research Implications

There was agreement among participants that salaried provider models were conducive to more proactive, multidisciplinary models of care. The perceived value of these forms of care is consistent with the broader evidence base within mainstream health services, where integrated models of care have been found to improve outcomes and reduce costs [11, 12]. However, a number of additional themes emerged that reflected the unique experiences of health providers within high-performance sport settings. These providers often have a responsibility for the overall health of a defined squad, or cohort of athletes, as opposed to a narrower focus on providing care to individuals. Consequently, the incorporation of continuous, preventative healthcare is recognised as an important component of a provider’s role, with the aim of reducing the burden of treatment provision in a commonly resource limited environment. While traditional indictors of athlete population health outcomes may align to those outside of sport (e.g. injury and illness incidence rates, health-related quality of life), the value of health practitioners working in sport may also be measured by individual athlete or team performance outcomes [13, 14]. This is likely in part because both individual and team performance is substantially impaired when the availability of athletes for selection is reduced due to injury and illness occurrence, which coincides with lower scoring and finishing positions across a range of sports [15].

To our knowledge, no previous studies have evaluated value-based reimbursement models within high-performance sport. However, there is an extensive body of literature from mainstream healthcare settings internationally [16–21]. Evidence for the effectiveness of these models is mixed, with several review papers noting that the relatively low quality of published evidence limits the ability to draw strong conclusions [18, 21–23]. However, it has been noted that the success of reimbursement models depends on an understanding of the context in which they are to be implemented [24, 25]; this may be supported by the active participation of providers in model design and development [25, 26].

### Strengths and Limitations

There are several strengths and limitations of this research. First, the focus group methodology was well suited to addressing the research question related to perceptions and experiences, but does not empirically quantify any of the phenomena discussed which was beyond the scope of the present study. Second, participants were purposively sampled to ensure a range of experiences and perspectives were represented, but participants did not necessarily have first-hand experience of each model discussed. Third, the insights presented here reflect those of the study participants only and cannot therefore be considered exhaustive. It is possible that additional insights, or differing viewpoints, may be held by individuals external to this study. Fourth, there were also athletes involved as participants in this study, who if included may also have contributed a valuable perspective as consumers of the health services being discussed. Fifth, participants were drawn from the high-performance sport community in Australia and findings may only be generalisable to similar high-performance sport communities.

## Conclusions

Overall, this study has highlighted the complex interplay of factors that may influence the implementation and effectiveness of value-based healthcare reimbursement models within high-performance sport. Our findings suggest that high-performance sporting organisations seeking to improve primary prevention and multidisciplinary care should consider the merits of salaried provider arrangements. A key recommendation arising from this research is that the future design and implementation of alternative payment models is informed by meaningful consultation with providers and other key stakeholders within the organisation. Future studies using rigorous experimental study designs are needed to test the effectiveness of alternative healthcare reimbursement models in high-performance sport settings.

## Supplementary Information

**Additional file 1**. Semi-structured question guide.

## Data Availability

Data within this study is unable to be made available for other studies due to the potential re-identifiability of participants. For specific requests, please contact the corresponding author.

## Abbreviations

EPIS: Exploration, Preparation, Implementation, Sustainment
FTE: Full-time equivalent
NSO: National Sporting Organisation
