# Supplementary material for: Alternative Reimbursement Models for Health Providers in High-Performance Sport: Stakeholder Experiences and Perceptions
Source: Sports Med Open. 2023 Jul 11;9:53. doi: 10.1186/s40798-023-00600-9 (PMC10335973; doi:10.1186/s40798-023-00600-9)
Supplement: Supplementary file 1 — Additional file 1. Semi-structured question guide. [file 40798_2023_600_MOESM1_ESM.pdf]

SUPPLEMENTARY FILE 1

Article title: Alternative reimbursement models for health providers in high performance sport: stakeholder experiences and perceptions

Journal name: Sports Medicine - Open

Authors

Hannah E. Carter, Michelle Allen, Liam A. Toohey, Steven M. McPhail, Michael K. Drew

Affiliations

Hannah Carter:

1. Australian Centre for Health Services Innovation and Centre for Healthcare Transformation, School of Public health and Social Work, Faculty of Health, Queensland University of Technology

Michelle Allen:

1. Australian Centre for Health Services Innovation and Centre for Healthcare Transformation, School of Public health and Social Work, Faculty of Health, Queensland University of Technology

Liam Toohey:

2. Athlete Performance Health, Australian Institute of Sport, Canberra
3. University of Canberra Research Institute for Sport and Exercise (UCRISE), Canberra

Steven M McPhail:

1. Australian Centre for Health Services Innovation and Centre for Healthcare Transformation, School of Public health and Social Work, Faculty of Health, Queensland University of Technology
2. Digital Health and Informatics Directorate, Metro South Health, Brisbane

Michael Drew

3. Athlete Performance Health, Australian Institute of Sport, Canberra
4. University of Canberra Research Institute for Sport and Exercise (UCRISE), Canberra

Corresponding author: Hannah E Carter: hannah.carter@qut.edu.au

HEALTH CARE FUNDING MODELS IN HIGH PERFORMANCE SPORT – SEMI-STRUCTURED WORKSHOP GUIDE

The following guide is intended to be used to conduct a semi-structured workshop for a project related to alternative health care funding models in high performance sport. This builds on the findings of a literature review on the same topic.

Objective:

To obtain key stakeholder insights into the acceptability, feasibility and sustainability of alternative health care funding models.

To identify factors that may support and barriers that may impede implementation of alternative funding models in the context of high-performance sport in Australia.

How to use this guide:

The questions are based on the constructs of the Exploration, Preparation, Implementation, Sustainment (EPIS) Framework[1] to ensure all aspects of implementation are considered in a comprehensive manner.

1. All key questions should be asked
2. Should other relevant issues be raised they will be explored in the relevant section, with prompting as required
3. Facilitators will ensure discussion progresses in a timely, yet informative manner.

[1] Aarons GA, Hurlburt M, Horwitz SM. Advancing a conceptual model of evidence-based practice implementation in public service sectors. Administration and Policy in Mental Health and Mental Health Services Research. 2011 Jan 1;38(1):4-23.

Introduction

- Introductions to the research team
- Overview of research question and aims and what we hope to achieve in this workshop
- Mention that session will be recorded, clarify issues relating to privacy and confidentiality and mention the opportunity to withdraw at any time
- Mention how this research will be used
- **Opportunity for participants to ask any initial questions**
- **Opportunity for participants to provide verbal consent**

Key topic 1: The innovation

A 20- 30 minute presentation will be given by a research team member to summarise preliminary findings from the literature review

Potential questions/ prompts

Let's start by discussing the services your [agency/group/organisation] currently delivers.

1. Tell us briefly about your experience delivering health services to high performance athletes and your [organisation's/group's] role in implementing these services.

2. Did you have to adapt your services to meet the contextual needs of this setting and characteristics of the patients? *(prompt: how might treating athletes be different than the general population??)*

3. Who is being provided for under the current service and who is missing out*? (prompt: who should be targeted if we were able to expand service provision more widely)*

4. How well do you believe current health services provision is working in terms of maximising athlete health and performance?

Key topic 2: Inner context facilitators and barriers

Let's talk specifically about the inner setting of your organisation/group now.

Potential questions/ prompts

What are the facilitators and barriers to implementation/service delivery in terms of:

1. **Leadership?**
   - Tell me about perceived support (or lack of) at all organisational levels for a potential change in health care funding or reimbursement models, away from fee for service?
   - Who would be critical to making implementation of new funding models a success? Consider individuals involved with oversight and/or decision-making who are proactive, knowledgeable, supportive and perseverant. *(prompt: who might block success?)*
2. **Characteristics of your organisation?**

- How does your organisation use new knowledge and support knowledge and skill sharing?
- What is the general level of receptivity in your organisation to implementing new change? Is there leadership, resourcing and support to do so? *(prompt: do organizational members feel committed to implementing an organizational change and confident in their collective abilities to do so?)*
- How does organisational culture, climate and communication impact service delivery? *(prompt: consider support for risk taking, teamwork, speed of action and tolerance of mistakes*
- Is adequate funding of health care service delivery a priority from the perspective of your organisation?

3. **Characteristics of people in your organisation?**
   - What shared or unique characteristics of individuals in your organisation influence the process of health service delivery (e.g. values, goals, attitudes, skills, social networks)? (prompt: what values regarding health and a good quality of life are highly regarded by members of your organisation)?

4. **Staffing processes?**
   - What processes or procedures are in place in the organisation related to the hiring, review, and retention of staff which impacts on health service delivery? *(prompt: staff turnover)*

Key topic 3: Outer context facilitators and barriers

Let's talk now about the outer setting of your organisation/group, considering its place in the broader high performance sport system in Australia

<u>**Potential questions/ prompts**</u>

What are the facilitators and barriers to implementation/service delivery in terms of:

1. **The service environment?**
   - What are the state and federal sociopolitical contexts that influence the process of implementation and service delivery? *(prompts: policies; legislation; monitoring and review; auditing; mandates)*

2. **Funding?**
   - Do you believe there is adequate resourcing currently available for health service provision?
   - Are there current incentives (or disincentives) that impact on service delivery and/or service change e.g. pay for performance schemes, alternative reimbursement mechanisms, grants, contracting arrangements etc.?

2. **Leadership?**
   - Who do you think are the key players at a system-wide level who would need to be involved in developing and implementing new funding models for health care provision, to give this the greatest chance of being successful?

3. **Patient/Client Advocacy?**

   Is service delivery at a system-wide level influenced by support or marketing for change by consumers i.e. athletes?

4. **Inter-organizational Environment & Networks**
   - Are there inter-organisational networks in which you are involved through which knowledge is shared and/or goals related to health care are developed? *(prompt: direct or indirect networking)*
   - Might these support or obstruct new service model changes?

5. **Patient/Client Characteristics**
   - Are their particular demographics and individual characteristics of the target population (i.e. athletes) that influence success or failure of service delivery?

Key topic 4: The innovation- Future directions?

And finally, just a couple of questions…

1. What services or activities have worked best to support athlete health and wellbeing in the past?
2. What is the single largest barrier or facilitator your [organisation/group] faces to being able to implement an alternative health care funding model?
3. What do you think is important for decision makers to know/understand about how to support the delivery of health services in high-performance sport at a system-level?
4. What do you see as being the ideal model for funding health care in a way that maximises outcomes from allocated resources?
